# Understanding the experiences of people with dementia and their family carers participating in healthcare student dementia education: A mixed-methods evaluation from the time for dementia programme

**DOI:** 10.1177/14713012231191412

**Published:** 2023-08-02

**Authors:** Stephanie Daley, Molly Hebditch, Yvonne Feeney, Georgia Towson, Joanna Pooley, Holly Pietersen, Sube Banerjee

**Affiliations:** Centre for Dementia Studies, 12190 Brighton and Sussex Medical School, University of Sussex, Brighton, UK; Faculty of Health, 6633 University of Plymouth, Plymouth, UK

**Keywords:** dementia education, undergraduate healthcare education, service user involvement, patient educators, lived experience, experts by experience

## Abstract

**Background:**

There is increasing awareness of the potential for positive impacts on student learning from involving people with dementia and family carers within undergraduate teaching. However, research on the experience of people with dementia and their family carers is sparse. This study aimed to evaluate the satisfaction and views of families (people with dementia and their family carers) who volunteered in Time for Dementia (TFD); an educational programme where undergraduate healthcare students visit families at home over a 2-year period.

**Methods:**

Families taking part in TFD completed a satisfaction survey after taking part in the programme (*n* = 803). Frequencies of satisfaction survey items were summarised and multiple linear regression models for factors associated with total satisfaction scores were produced. Open text responses were analysed using thematic framework analysis as to the most favourable aspects of the programme and areas requiring improvement.

**Results:**

Overall satisfaction was high for taking part in TFD, with a perception of contribution, and being of value. There was strong evidence that families enjoyed the experience and would recommend participation to others. Higher numbers of student visits were significantly associated with greater satisfaction. Families identified aspects of the programme that benefited them, with social interaction rating highly. Improvements suggested by the families included increased visit structure and organisational improvements.

**Conclusions:**

This study has sought to evaluate at scale the satisfaction of families taking part in a dementia education programme. It is positive that families report high satisfaction in the programme and identify perceived value for themselves as well as students, suggesting reciprocal benefits. This study contributes to the broader understanding of what Experts by Experience value when taking part in educational interventions.

## Background

It is widely accepted that the quality of healthcare for people with dementia is sub-optimal and requires substantial improvement (World Health Organization, 2017) with over 29 countries developing national action plans in response to this challenge (Chow et al., 2018; Department of Health, 2015). The priority for action is further driven by the growing numbers of people with dementia, with 46 million people estimated to have dementia globally, with a predicted doubling in the next 20 years (World Health Organization, 2015).

A core component of enhancing dementia care is by improving the dementia knowledge, skills and attitudes of the health and social care workforce through tailored education (Alzheimer’s Disease International, 2019; Department of Health, 2009). Dementia education is needed for all healthcare professionals, regardless of specialism, as people with dementia access a range of healthcare services, not just dementia or geriatric services, due to high levels of multimorbidity (Banerjee, 2015; Bunn et al., 2014). An important but under-leveraged area for attention is the undergraduate curriculum during training (Alushi et al., 2015; Pulsford et al., 2007; Tullo and Gordon, 2013). Traditional, lecture-or placement-based, undergraduate healthcare education has been criticised for creating a narrow view of dementia, which constrains skill development (Banerjee et al., 2017; Tullo and Gordon, 2013).

It is in this context that a range of innovative dementia education interventions have been developed (Banerjee et al., 2017; Goldman and Trommer, 2019; Jefferson et al., 2012; Mastel-Smith et al., 2020; Wood et al., 2016). Such programmes aim to address specific gaps in undergraduate dementia education, including an understanding of person-centred practices, empathy development, and the improvement of comfort with and positive attitudes towards working with people with dementia. A recent review suggests there is preliminary evidence that educational programmes are most effective when they directly involve people with dementia (Williams and Daley, 2021). This perhaps is not surprising since the active involvement of ‘Experts by Experience’^1^ have long been valued in healthcare education particularly as a means of developing a person-centred focus (Gordon et al., 2020; Towle et al., 2010).

Time for Dementia (TFD) is an innovative long-term experiential programme set up in the UK in 2014. The programme consists of students meeting a family (a person with dementia and their carer) for 2 hours at the family’s home, up to 6 times over 2 years, supported by other learning activities (Banerjee et al., 2017). TFD is novel in its size and scope; it is a mandatory component of the healthcare professionals' course which has, to date, been delivered to over 6500 medical, nursing, paramedic, and allied health profession students at seven universities in England. Results of the evaluation of the programme suggest positive impacts on students’ knowledge, attitudes, and practice (Banerjee et al., 2021; Daley et al., 2020; Grosvenor et al., 2021; Daley et al, 2023).

The success of such programmes as TFD relies on families (people with dementia and their family carers) to volunteer their time to contribute to the education of future healthcare professionals. At a minimum, participating in the programme should not cause harm or distress, but ideally it should also be enjoyable or satisfying. This is important ethically but is also necessary to recruit and retain the necessary number of volunteer families to deliver the programme.

The current evidence about the experience of people with dementia and their carers contributing to undergraduate educational programmes is limited and predominantly consists of small qualitative studies. Key themes include the enjoyment of interacting with students (Annear et al., 2017; Han and Radel, 2017; Morhardt et al., 2019; Russell, 2020) and contributing to society (Annear et al., 2017; Han and Radel, 2017; Russell, 2020). These themes are also reflected in a qualitative study of the early development of TFD which identified that wanting to contribute to student learning was a motivating factor in participation, whilst enjoyment of the visits was a sustaining factor, and wider benefits to families were identified (Cashin et al., 2019).

This study was undertaken to explore families' experiences further, by evaluating quantitatively and qualitatively the satisfaction and views of the families participating in TFD and answer the following research questions: (i) how satisfied were the families involved in TFD; and (ii) what factors are associated with satisfaction in TFD.

## Methods

### Design

This is an analysis of quantitative and qualitative data collected from 2015 to 2021 as part of the TFD evaluation (Banerjee et al., 2017) which has the form of a cohort study. Data were collected from families before their first student visit (baseline) and approximately 24 months later (follow up) after students had completed the programme.

### Study setting and sample

Participants took part in the research as a dyad consisting of a person with dementia and a family carer, (hereafter referred to together as ‘families’). Inclusion criteria were that they were recruited to take part in the TFD programme between 2015 and 2020. Some families have, in series, hosted more than one set of students, these data are taken only from their first set of student visits.

Families hosted visits with undergraduate students from medicine, nursing, paramedic, or allied health professions (occupational therapy, physiotherapy, speech and language therapy and radiography) at five universities covering the South of England. All families received face-to-face visits in their own homes with students, however from March 2020 due to the COVID-19 pandemic, in-person student visits were halted and telephone calls replaced follow up visits for those with outstanding visits.

### Measures

Baseline measures included demographics for family carers and the person with dementia. Demographic data were chosen to aid understanding of the characteristics of the sample and variety in terms of carer relationships and living situations, including factors that may relate to volunteering behaviours (e.g. working status).

Follow up measures included a satisfaction survey and the number of visits hosted by the family. The Standardized Mini-Mental State Examination (sMMSE) was completed by participants with dementia at both time points as an assessment of cognition and dementia severity (Molloy and Standish, 1997).

The satisfaction survey was created for this study with the inclusion of 8 items adapted from the patient and carer evaluation used in The Buddy Program (Morhardt, 2006), a similar longitudinal dementia educational programme. There were two versions of the survey with items tailored for the person with dementia or carer. Both included 13 items designed to assess satisfaction with the ‘organisation’ of the programme (Q1,3,4,8), and ‘comfort and enjoyment’ of student visits (Q2,5,6,7,9,10,11,12,13). Overall satisfaction was rated using Likert scales ranging from 1 = strongly disagree, to 5 = strongly agree. Both surveys also had two open text questions:

• “What were the BEST aspects of the Time for Dementia programme?”
• “What IMPROVEMENTS could be made to the Time for Dementia programme?”

### Procedure

The charitable organisation Alzheimer’s Society managed the recruitment and enrolment of family volunteers to the TFD programme. During induction to the TFD programme, families were additionally invited to take part in the linked research study. Families who expressed an interest in the research were approached by the researchers over the telephone and study information was sent via email or post. Written or verbal consent was obtained and recorded for all participants, and capacity to consent with the person with dementia was assessed by a trained researcher. NHS Health Research Authority Ethics approval was obtained from London - Queen Square Research Ethics Committee. (REC ref: 15/LO/0046).

For families recruited in the period 2015 to 2017, consent and measures were completed by a researcher at the participants' homes. In 2018, the study protocol was changed, and consent and measures were completed over the telephone due to the expansion of the programme. Due to the inability to assess capacity to consent by telephone with people with dementia, the study from this point onwards only included carer participants. The two time periods are classified as Phase 1 (2015–2017) which includes people with dementia and their carers and Phase 2 (2018–2021) which includes only carers.

### Analysis

Summary statistics are reported for demographic information and frequencies of responses for satisfaction items.

Thematic framework analysis (Ritchie and Spencer, 1994) was completed for the participant responses to open text questions for the best aspects of the programme and suggested improvements. The framework was developed following a previous analysis of interviews with TFD families (Cashin et al., 2019). Three researchers (YF, HP and JP) coded the transcripts using Excel (v1808) under the supervision of an experienced qualitative researcher (SD). Each meaningful unit of text was coded with a descriptive code.

Total satisfaction scores for the two survey sections (organisation, and comfort and enjoyment) for both people with dementia and family carer were calculated. Satisfaction surveys were excluded if more than 20% of items were missing in each subsection, otherwise person-mean imputation was used. Total scores had acceptable reliability for three of the four sections (Cronbach’s α .64–.85), the reliability for total satisfaction score for ‘organisation’ as rated by people with dementia was low (.26) and therefore the associated results should be interpreted with caution. In a multiple regression model factors that predicted satisfaction in family carers and people with dementia were explored for both subsections of the satisfaction survey. For people with dementia, possible predictors included sex (male vs. female), age, sMMSE score at follow up (continuous), and the number of visits. Possible predictors for carers included: sex (male vs. female), age, relationship to the person with dementia (spouse/partner vs. other), number of visits, and TFD phase (1 vs. 2). Statistical significance was defined as a *p*-value <.05. All data were analysed using SPSS V.26.

## Results

### Response rate

1,061 families were eligible to take part in the study. 803 (69%) families consented to take part and completed baseline measures (this included 400 in Phase 1 and 403 in Phase 2). Of this, 442 (55%) contributed to follow up data (208 in Phase 1 and 234 in Phase 2),with 421 families responding to the open text questions on the survey.

### Demographics

The characteristics of families are provided in Table 1. The majority of carers were female (67%), spousal carers (81%), with a median age of 73 years (23–90). People with dementia were mostly male (58%), living in the community (99.2%), with a median age of 79 years (52–104).

**Table 1.** Baseline family characteristics (*n* = 803).

	Person with dementia		Carer	
	Median	IQR	Median	IQR
**Participant’s age**	79	73.0 to 84.0	73	66.0 to 79.0
	**No.**	**%**	**No.**	**%**
**Participant’s gender**				
Male	462	57.5	269	33.5
Female	341	42.5	534	66.5
Total	803	100.0	803	100.0
**Participant’s ethnicity**				
White British/European	784	98.0	778	98.9
Mixed/Multiple ethnic groups	2	0.3	1	0.1
Asian/Asian British	2	0.3	2	0.3
Black/African/Caribbean/Black British	2	0.3	0	0.0
Other	10	1.3	6	0.8
Total	800	100.0	787	100.0
**Participant’s highest education level**				
Less than primary school	1	0.1	3	0.4
Primary school completed	54	6.8	28	3.5
Secondary school completed	462	58.3	409	51.5
College completed	119	15.0	137	17.3
University completed	107	13.5	138	17.4
Post graduate degree completed	50	6.3	79	9.9
Total	793	100.0	794	100.0
**Participant’s current working status**				
Yes	13	1.6	136	17.2
No	788	98.4	657	82.8
Total	801	100.0	793	100
**Participant’s diagnosis**				
Alzheimer’s disease	356	45.1	-	-
Mixed diagnosis	194	24.6	-	-
Early onset dementia	29	3.7	-	-
Dementia unspecified	33	4.2	-	-
Vascular dementia	103	13.0	-	-
Frontotemporal (and Pick’s disease)	27	3.4	-	-
Parkinson’s Disease dementia	15	1.9	-	-
Posterior cortical atrophy	6	0.8	-	-
Dementia with lewy Bodies	26	3.3	-	-
Other	1	0.1	-	-
Total	790	100.0	-	-
**sMMSE category**				
May be normal (30–26)	89	23.7	-	-
Mild/early (25–20)	163	43.5	-	-
Moderate (19–10)	103	27.5	-	-
Severe (9–0)	20	5.3	-	-
Total	375	100	-	-
**Living situation of person with dementia**				
Community	604	99.2	-	-
Residential care facility	12	0.8	-	-
Total	616	100.0	-	-
**Carer’s relationship to person with dementia**				
Spouse/Partner	-	-	644	80.5
Other (e.g. child, sibling)	-	-	156	19.5
Total	-	-	800	100.00

Abbreviations: sMMSE: Standardized Mini-Mental State Examination.

### Satisfaction

**Figure 1.**
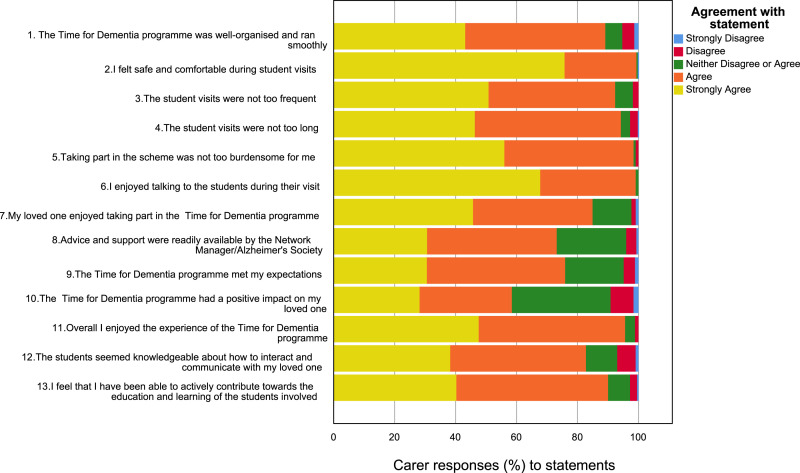
Carer satisfaction (*n* = 415).

**Figure 2.**
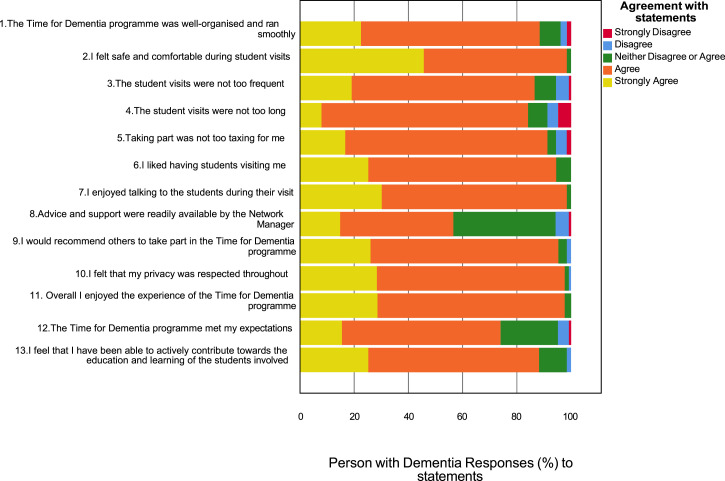
People with dementia satisfaction (*n* = 122).

Overall satisfaction with participation in the programme was high. The most common response was ‘strongly agree’ or ‘agree’ for each item on the satisfaction survey. Responses to items are provided in Figures 1 and 2.

88% of people with dementia and 90% of carers either ‘strongly agreed’ or ‘agreed’ that they had been able to actively contribute towards the education and learning of the students involved. 98% of people with dementia and 96% of carers either ‘strongly agreed’ or ‘agreed’ that they had enjoyed the experience of the TFD programme. 59% of carers indicated a positive impact for their loved one taking part and 95% of people with dementia agreed that they would recommend others to take part in TFD.

### Predictors of satisfaction

Follow up data is presented in Table 2. This includes total satisfaction scores for the person with dementia and carer, the total number of student visits, and the severity of dementia.

**Table 2.** Family Follow up measures.

Family outcome	Mean	SD	*n*
**Number of student visits hosted by family**	4.5	2.0	408
**Satisfaction total scores**			
Carer - organisation (4–20)	17.0	2.1	414
People with dementia - organisation (4–20)	15.5	1.8	122
Carer - comfort (9–45)	38.8	4.5	415
People with dementia - comfort (9–45)	37.6	3.3	123
**sMMME total score (person with dementia)**	18.4	6.8	165
**sMMSE category (person with dementia)**	**No.**	**%**	
May be normal (30–26)	25	15.2	
Mild/early (25–20)	63	38.2	
Moderate (19–10)	57	34.5	
Severe (9–0)	20	12.1	
Total	165	100.0	
**University of visiting students**			
Brighton and Sussex Medical School	121	27.4	
University of Surrey	141	31.9	
University of Brighton	51	11.5	
University of Greenwich	34	7.7	
Canterbury Christ Church University	95	21.5	
Total	442	100.0	

## Organisation

Table 3 presents the results of the multiple regression models for predictors of satisfaction with the organisation of the programme. For carers, there was strong evidence of an association with higher satisfaction and higher number of visits (coefficient: .18, 95% confidence interval [95% CI]: .07 to .29, *p* < .001), and taking part in Phase 1 (−.59, 95% CI: −1.03 to −.15, *p* = .009). There was no evidence to support an association with carer age, gender, or carer relationship. For people with dementia, there was also evidence for a positive association with the number of visits (.21, 95% CI: .03 to .38, *p* = .022). No association was found with participant age, gender, or sMMSE score.

**Table 3.** Predictors of satisfaction with Organisation.

Variables	B	Lower 95% CL	Upper 95% CL	*p*
Family carers (*n* = 396)				
(Constant)	16.30	13.26	19.34	<.001
Age of carer	.01	−.02	.03	.745
Gender of carer (male vs. female)	.21	−.24	.67	.353
Carer relationship to participant (spouse/Partner vs. other)	.11	−.61	.84	.756
TFD phase (1 vs. 2)	−.59	−1.03	−.15	.009
Total number of student visits	.18	.07	.29	<.001
**People with dementia (*n* = 107)**				
(Constant)	15.73	11.63	19.84	<.001
Age of participant	−.02	−.06	.03	.383
Gender of participant (male vs. female)	.35	−.30	1.01	.286
sMMSE score	−.02	−.07	.04	.565
Total number of student visits	.21	.03	.38	.022

sMMSE: Standardized Mini-Mental State Examination, TFD: Time for Dementia. Dichotomous variables are coded 0 versus 1.

## Comfort and enjoyment

Table 4 presents the results of the multiple regression models for predictors of satisfaction with comfort and enjoyment of the programme. There was strong evidence of an association with higher satisfaction and a higher number of visits (.69, 95% CI: .47 to .91, *p* < .001), and some evidence for female gender (1.09, 95% CI: .18 to 1.99, *p* = .019). There was no evidence to support an association with carer age, TFD Phase, or carer relationship. For people with dementia, there was evidence that younger age was associated with greater satisfaction (−.09, 95% CI: −.18 to −.01, *p* = .028) and weak evidence for the number of visits (.32, 95% CI: −.01 to .65, *p* = .059). No association was found with participant gender or sMMSE scores.

**Table 4.** Predictors of satisfaction for comfort and enjoyment.

Variables	B	Lower 95% CL	Upper 95% CL	*p*
**Family carers (*n* = 396)**				
(Constant)	35.85	29.79	41.90	<.001
Age of carer	−.02	−.08	.03	.454
Gender of carer (male vs. female)	1.09	.18	1.99	.019
Carer relationship to participant (spouse/Partner vs. other)	.02	−1.42	1.46	.980
TFD phase (1 vs. 2)	−.36	−1.24	.53	.428
Total number of student visits	.69	.47	.91	<.001
**People with dementia (*n* = 107)**				
(Constant)	41.91	34.20	49.62	<.001
Age of participant	−.09	−.18	−.01	.028
Gender of participant (male vs. female)	.45	−.78	1.67	.470
sMMSE score	.04	−.07	.14	.515
Total number of student visits	.32	−.01	.65	.059

sMMSE: Standardized Mini-Mental State Examination, TFD: Time for Dementia. Dichotomous variables are coded 0 versus 1.

### Best aspects and improvements

Quotes from the qualitative feedback from open text questions are presented in Table 5. Responses were coded into seven main categories for best aspects of the programme: *social interaction* (42%), *feelings of making a difference* (34%), *student behaviour* (17%), *organisation of the programme* (3%), *unable to identify best aspects (*2%), *feedback* (1%), and *COVID- keeping in touch* (1%). *Social interaction* included how family carers identified TFD as an enjoyable experience. Meeting new people and, in particular, young people was enjoyable and social benefits for the person with dementia were noted. *Feelings of making a difference* included the perception that students were learning from families. Positive *student behavior* included appreciation for ‘friendly’, ‘nice’, ‘pleasant’ students who showed interest and engagement.

**Table 5.** Quotes from families.

Best aspects
** Social interaction**
*‘It was nice for* [person with dementia] *to see them and join in the conversation. It was also quite therapeutic for me* [a carer] *to talk and it was great to have younger people come to chat to us, so that was delightful.’*
*‘My mum really enjoyed chatting to the students, she got a lot out of it. It was interesting for me to see mum interacting with others.’*
**Making a difference**
‘*The opportunity to talk to young professionals in the relaxed situation of our home, with time to express our thoughts and listen to their views and comments, we value the aim of the programme to include us in this training initiative.’*
**Student behaviour**
*‘Two intelligent people who appeared to listen and be interested in our experience’*
**Improvements**
**Structure of visits**
*‘I question how much they got out if it. They were not very structured in their approach, and they didn’t have much of a plan and it was more chit chat.’*
**Organisation**
‘…*not a regular thing, only been 2 occasions and too too many months apart*.’
**Feedback**
*‘More feedback on what the students get out of the visit.’*
**Student behaviour**
*‘Students could read their pre-arrival information.’*

Six main categories were identified for improvements: *no improvements* (25%), *the structure of visits* (22%), organisation of the *programme* (20%), *feedback* (13%), *student behaviour* (11%), *COVID- visits interrupted* (5%), and *unable to identify improvements* (4%). Suggested improvements for the *structure of visits* included more guidance to students on preparing for the visits and encouraging direct questioning from students around dementia. *Organisation of the programme* included issues with arranging student visits, communication problems with students and universities, as well as comments on the number and frequency of visits; most commonly suggesting more visits. Families also felt that they would like *feedback* about student learning. *Student behavior* they perceived as needing improvement included lack of preparation and lack of interest and engagement by students.

## Discussion

### Key findings

This is the first large scale evaluation of the satisfaction of people with dementia and their carers taking part in a dementia education intervention. The results suggest that the families completing TFD have a high level of satisfaction (Figures 1 and 2). Importantly there were no indications of harm. There is also some evidence that an increased number of visits is associated with greater satisfaction (Tables 3 and 4). Previous qualitative work on TFD found that families valued the long term continuity of visits and saw this as important to building relationships with students (Cashin et al., 2019). The importance of these relationships is also reflected in student accounts of relational learning being a key factor influencing positive learning (Daley et al., 2020; Grosvenor et al., 2021). The value of longitudinal contact is further supported by the qualitative analysis which indicates that some families would have liked more visits. Taken together these data provide encouraging support for dementia education programmes such as TFD.

It is encouraging that people with dementia and family carers identified positive impacts from taking part in TFD for themselves. Though the primary aim of the programme is to educate students, it is positive it is not seen as a one-sided relationship. Specifically, families described feelings of therapeutic social interaction, which is theorised to be a central benefit in other longitudinal educational programmes (Han and Radel, 2016, 2017; Morhardt, 2006). Furthermore, families derived satisfaction from feelings of making a difference. Knowingly contributing to healthcare education may promote personhood and wellbeing in people with dementia through positive feelings associated with their ongoing contribution to society and advocacy (Annear et al., 2017; Russell, 2020; Weetch et al., 2021). Identified improvements by families suggest this could be strengthened by giving families feedback on what students gained from their contributions.

The finding that higher levels of satisfaction in the organisation of the programme were associated with Phase 1, could be interpreted as a function of COVID-19 disruption in Phase 2. COVID-19 interruption reduced the number of face-to-face visits with families, and included a degree of general disruption to the administration of the programme, due to the wider disruption across higher education.

There was some evidence to suggest that female carers and younger people with dementia may be more satisfied with the programme but this small effect does not suggest a disproportionate difference in experience. People of both sexes and all ages reported high levels of satisfaction. There were no key characteristics that influenced satisfaction of concern to make recommendations on modifying recruitment strategies.

The results indicate that there may be differences in satisfaction between people with dementia and carers. For example, the significant association of visit number with comfort and enjoyment for carers but not for people with dementia. However, any differences between the satisfaction of carers and people with dementia could be due to the scales consisting of different items (so not directly comparable), differences in response rates or comprehension. Therefore, we caution against direct comparisons.

The results suggest what families valued from their participation in a dementia education programme, and provides insights that can enhance further development. e.g., the family’s expectations of delivery (e.g. the number of visits and organisation) and how students structure their visits affects a family’s satisfaction. This points to the importance of students' and families' expectations being aligned from the beginning, starting with the information that each side receives. These findings have been fed into the iterative development of TFD allowing for more prescriptive guidance to students about how to structure and plan their visits Furthermore, individual modification of the programme may be required to increase satisfaction. For instance, a number of people with dementia disagreed that the visits ‘were not too long’ suggesting some may prefer shorter visit duration. This suggests that there needs to be clearer messaging to students to ensure that mutual expectations, say about visit length, are discussed and agreed at the beginning of each visit.

### Strengths and limitations

The main limitation is the possibility of non-response bias caused by loss to follow up. This loss to follow-up was primarily from those leaving the TFD programme for reasons including death, change in health and family circumstances. Participation in TFD was voluntary, and reasons for withdrawal were not required and not consistently recorded. Therefore, because the satisfaction survey was only collected for those who completed the programme, the results may include a survivor bias. Furthermore, the lower response rate for people with dementia was likely to be due to cognitive impairment as well as not being eligible to take part in Phase 2. There were also limitations on the wording of the survey, e.g., double negatives and lack of clarity of the terms (i.e. ‘network manager’) may have made some items unclear. Also, due to issues of recall, a post hoc satisfaction survey or interview may not be an optimal method of gaining feedback from people with dementia (Bartlett, 2012). Further research has since been completed, with lived experience input, looking at alternative methods of obtaining feedback from people with dementia in TFD using video recordings of visits. Lastly, COVID-19 disruption at the time of data collection added complexity to drawing interpretations from these results.

The main strength of the study is that it adds to the limited literature on the experiences of Experts by Experience in dementia educational programmes. In addition, it includes a large number of participants and has generated qualitative as well as quantitative data together to provide a broad assessment of the programme from the family viewpoint. Finally, in Phase 1 we were inclusive of the perspective of the person with dementia which is often not voiced in such evaluations.

### Conclusion

The overall satisfaction of families completing TFD was high. Families attributed social interaction and feelings of making a difference to students as some of the best aspects of the programme. Issues with administration, expectations on visit structure and preparation from students may have limited satisfaction. These results suggest families strongly value the TFD programme, both for students and themselves. Future research should explore the active benefits to families, the experiences and reasons of withdrawal, and the experiences over time for those who continue in the programme.

## Note

1. It is recognised that a variety of terms are used for this role, namely, Patient Educators, Experts by Experience, or Lived Experience input - for this paper we will use the term ‘Experts by Experience’ since this was preferred by our participants.

## Appendix

### Abbreviations

TFD: Time for dementia

### Anonymous identifiers

HP: Holly Pietersen
JP: Joanna Pooley
SD: Stephanie Daley
YF: Yvonne Feeney
